# Risk factors for genital infections in people initiating SGLT2 inhibitors and their impact on discontinuation

**DOI:** 10.1136/bmjdrc-2020-001238

**Published:** 2020-05-24

**Authors:** Andrew P McGovern, Michael Hogg, Beverley M Shields, Naveed A Sattar, Rury R Holman, Ewan R Pearson, Andrew T Hattersley, Angus G Jones, John M Dennis, MASTERMIND consortium, William E Henley

**Affiliations:** 1 University of Exeter Medical School, Institute of Biomedical and Clinical Science, University of Exeter, Exeter, Devon, UK; 2 Institute of Cardiovascular Sciences, University of Glasgow, Glasgow, UK; 3 Radcliffe Department of Medicine, University of Oxford, Oxford, Oxfordshire, UK; 4 Division of Population Health & Genomics, School of Medicine, University of Dundee, Dundee, UK

**Keywords:** non-insulin treated type 2 diabetes, candida, A1C, adherence to medications

## Abstract

**Introduction:**

To identify risk factors, absolute risk, and impact on treatment discontinuation of genital infections with sodium-glucose co-transporter-2 inhibitors (SGLT2i).

**Research design and methods:**

We assessed the relationship between baseline characteristics and genital infection in 21 004 people with type 2 diabetes initiating SGLT2i and 55 471 controls initiating dipeptidyl peptidase-4 inhibitors (DPP4i) in a UK primary care database. We assessed absolute risk of infection in those with key risk factors and the association between early genital infection and treatment discontinuation.

**Results:**

Genital infection was substantially more common in those treated with SGLT2i (8.1% within 1 year) than DPP4i (1.8%). Key predictors of infection with SGLT2i were female sex (HR 3.64; 95% CI 3.23 to 4.11) and history of genital infection; <1 year before initiation (HR 4.38; 3.73 to 5.13), 1–5 years (HR 3.04; 2.64 to 3.51), and >5 years (HR 1.79; 1.55 to 2.07). Baseline HbA_1c_ was not associated with infection risk for SGLT2i, in contrast to DPP4i where risk increased with higher HbA_1c_. One-year absolute risk of genital infection with SGLT2i was highest for those with a history of prior infection (females 23.7%, males 12.1%), compared with those without (females 10.8%, males 2.7%). Early genital infection was associated with a similar discontinuation risk for SGLT2i (HR 1.48; 1.21–1.80) and DPP4i (HR 1.58; 1.21–2.07).

**Conclusions:**

Female sex and history of prior infection are simple features that can identify subgroups at greatly increased risk of genital infections with SGLT2i therapy. These data can be used to risk-stratify patients. High HbA_1c_ is not a risk factor for genital infections with SGLT2i.

**Significance of this study**

**What is already known about this subject?**

- It has been established that sodium-glucose co-transporter-2 inhibitors (SGLT2i) are associated with greater risk for genital infections. However, patient features which confer the greatest risk are not well elucidated. Female gender is a known risk factor.

**What are the new findings?**

- Prior history of genital infection and gender are the two main determinants of risk of genital infection with SGLT2i.
- High HbA1c does not increase the risk of genital infection in those starting an SGLT2i, in contrast to those starting a DPP4 inhibitor.
- Genital infections are associated with only a slight increase in treatment discontinuation.

**How might these results change the focus of research or clinical practice?**

- These data can be used by clinicians to estimate the infection risk for individual patients and hence support more informed decision making.

## Introduction

Sodium-glucose co-transporter-2 inhibitors (SGLT2i) are an increasingly important oral medication class in type 2 diabetes^1–3^ with their use climbing dramatically in recent years.^4–7^ They result in a broadly similar amount of glucose lowering compared with other oral agents but can also reduce blood pressure and result in modest weight loss.^8–10^ In addition to their glucose lowering effects, large-scale clinical trials have demonstrated reduction in cardiovascular and renal outcomes in high-risk groups with type 2 diabetes,^11–13^ as well as benefit in patients with heart failure whether or not they have type 2 diabetes.^14^ They can also be used as adjuvant therapy to insulin for the treatment of type 1 diabetes.^15^

SGLT2i reduce hyperglycemia in people with diabetes by increasing urinary excretion of glucose.^8 16^ This induced glycosuria increases the risk of genital infections^16^ and both clinical trials and observational studies demonstrate a 2.5–6-fold increase in genital infections in people using SGLT2i compared with controls.^10 16 17^ A number of factors have been shown to be associated with risk of genital infection in the general population, in particular, female sex and diabetes, especially when glycemic control is poor.^18 19^ However, there has been limited investigation of the risk factors for genital tract infection in those initiating SGLT2i therapy, or of the impact of infection on treatment discontinuation outside of a trial setting.

We aimed to determine the factors associated with the risk for developing a genital infection while on SGLT2i treatment and the impact of these infections on treatment discontinuation.

## Research design and methods

We conducted a retrospective cohort analysis of people with type 2 diabetes initiating SGLT2i within a large population-based UK cohort; the UK Clinical Practice Research Datalink (CPRD). We examined the prevalence of genital infections during the first year of treatment. We explored the associations between baseline characteristics and history of previous genital infections on infection risk during treatment and examined the impact of genital infections occurring early during treatment on subsequent medication discontinuation. For all analyses, we used people initiating dipeptidyl peptidase-4 inhibitors (DPP4i) as a comparison cohort. We used all available data up to the point of data extraction, July 2019.

### Setting and participants

CPRD is one of the larger longitudinal population-based medical records datasets in the world and provides a representative sample of the UK population.^20^ People with type 2 diabetes were identified using a method we have previously described in detail.^21^ In summary, they were identified using the presence of a diagnostic code for diabetes and the prescription of one or more glucose lowering medication(s). People were excluded if they had possible type 1 diabetes or another diabetes type. Possible type 1 diabetes was defined as age of diagnosis less than 35 years, treatment with insulin therapy only, or initiation of insulin within 1 year of diagnosis. Other forms of diabetes were excluded using the presence of indicative diagnostic codes including steroid-induced diabetes, gestational diabetes and monogenic diabetes.

The date of diabetes diagnosis was defined as the date of the first of: a diabetes diagnostic code, glucose lowering medication, or glycated hemoglobin (HbA_1c_)≥6.5% (48 mmol/mol). Those who had a first diabetes indicator within 3 months of practice registration were excluded as their diagnosis date was uncertain and treatment history could not be determined.

From the cohort of people meeting our criteria for type 2 diabetes with an identifiable date of diagnosis, we included everyone initiating SGLT2i (canagliflozin, dapagliflozin, and empagliflozin). This group then formed our main study cohort. We identified a comparison group of all people initiating DPP4i.

### Participant baseline characteristics

We report the baseline clinical characteristics of both treatment groups at medication initiation; age, sex, duration of diabetes, and body mass index (BMI), HbA_1c_, estimated glomerular filtration rate (eGFR), history of genital infections. BMI, HbA_1c_, and eGFR were defined using the most recent measurement within the 2 years prior to the drug start date. We excluded people with missing data for these baseline characteristics or with a baseline eGFR <45 mL/min.

### Outcomes

A genital infection was defined as either a diagnosis code specific for a genital infection (eg, candida vaginitis or vulvovaginitis in women, or balanitis, balanoposthitis in men), a prescription for antifungal therapy used specifically to treat genital infections (eg, an antifungal vaginal pessary), or a non-specific diagnosis of “thrush” with a topical antifungal prescribed on the same day. The timing of the most recent infection prior to the start of the index drug was used as a baseline variable and categorized as; occurring within a year, between 1 and 5 years, or longer than 5 years before start of the index drug.

Treatment discontinuation was defined as no repeat prescription issue of the index drug for 3 months (90 days). The discontinuation date was defined as 90 days after the date of the last prescription issue.

### Statistical methods

Associations between baseline characteristics and time to genital infection were assessed using Kaplan-Meier survival plots. We then performed multivariable Cox-regression analyses of time to genital infection separately for SGLT2i-treated and DPP4i-treated patients. Multivariable models were defined a priori to include age, sex, duration of diabetes, HbA_1c_ prior to treatment, eGFR, BMI, and previous history of genital infections. People with incomplete follow-up were included and censored at loss to follow-up. Time to infection models was also censored at discontinuation of the medication of interest (SGLT2i or DPP4i). Drug additions or switching of background medications was ignored. Complete available follow-up data were used for the Cox models, that is, where follow-up beyond the first year of treatment was available, these data were also included in the models.

As a key baseline variable of interest, we also evaluated the non-linear association between HbA_1c_ prior to drug initiation and subsequent risk for genital infection by fitting continuous baseline HbA_1c_ as a restricted cubic spline with three knots, with adjustment for the same factors used in the multivariable models.

Based on the results of Cox regression models, we defined four important clinical risk groups using the two most discriminative baseline features: sex, and history of one or more genital infection. For each risk group, we report the proportion of people developing a genital infection during the first year of treatment. We also report the annual cumulative incidence of infection out to 4 years, in each risk group.

The impact of early infection on discontinuation was assessed using multivariable Cox-regression separately for SGLT2i-treated and DPP4i-treated patients. Multivariable models were adjusted for the same baseline variables included in the infection risk models: age, gender, duration of diabetes, HbA_1c_ prior to treatment, eGFR, BMI, and previous history of genital infections.

### Sensitivity analyses

Patient characteristics influence treatment selection and therefore potentially influence the results of our primary analysis. To explore this possibility further, we repeated the primary analysis in propensity score-matched subgroups. Matching by treatment choice (SGLT2i or DPP4i) was performed using a 1:1 matching ratio and a nearest neighbor matching algorithm (R MatchIt V3.0.2). Variables included in the propensity matching were: age, sex, duration of diabetes, number of concurrent medications, HbA_1c_ prior to treatment, eGFR, BMI, and previous history of genital infections. We compared the baseline characteristics between the SGLT2i and DPP4i matched groups using the χ² test for categorical variables and the unpaired t-test for continuous data. All reported p values are two-sided. We used these matched cohorts to replicate the primary analysis for genital infection risk. We also used them to examine the impact of treatment selection on genital infection rates overall and in our four clinical risk groups, that is, we compare the additional risk for infection incurred by treatment with an SGLT2i compared with a DPP4i.

Posthoc, we also performed two additional analysis. The first, a sensitivity analysis, explored the impact of removing genital infections identified using a combination of the non-specific diagnosis code “thrush” and a simultaneous topical treatment. This sensitivity analysis was only performed in females, as there was no condition specific treatment available (eg, antifungal pessaries) for males and as there were fewer diagnosis specific codes in males compared with females. The second explored associations between concurrent medications with potential associations with fungal infection: corticosteroids,^22^ oestrogen therapies,^23 24^ and non-steroid immune-modulating medications.^25^ These were defined as being prescribed at baseline if a prescription was present in the clinical record within the 3 months prior to initiation of the index drug. All oral corticosteroids were included for analysis; topical or other preparations were not considered. Oestrogen therapies comprised hormone replacement therapies, combined oral contraceptives, and topical vaginal oestrogen preparations. Immune-modulating medications comprised all non-steroid immunosuppressants listed in the British National Formulary including thiopurines (azathioprine and mercaptopurine), methotrexate, calcineurin inhibitors (tacrolimus and cyclosporine) and monoclonal antibodies.

## Results

Within CPRD, we identified 302 857 people who met our criteria for type 2 diabetes. Of these, 22 928 were initiated on an SGLT2i and 67 104 on a DPP4i. There was incomplete baseline data on HbA_1c_, eGFR, or BMI for 1920 (8.4%) people initiating an SGLT2i and 11 623 (17.3%) initiating a DPP4i. There were some baseline differences between the remaining cohorts; those initiating SGLT2is were younger, had a higher male proportion, longer duration of diabetes, higher HbA_1c_, eGFR, and BMI, and more previous genital infections (table 1). The SGLT2i and DPP4i most commonly initiated were dapagliflozin and sitagliptin, respectively (online supplementary table S1).

10.1136/bmjdrc-2020-001238.supp1 Supplementary data

**Table 1:** The baseline characteristics of people initiated on an SGLT2i or DPP4i

	SGLT2i (n=21 008)	DPP4i (n=55 481)
Age (years)	60.4 (9.3)	63.3 (10.8)
Female (n (%))	8115 (38.6)	22 274 (40.1)
Duration of diabetes (years)	9.1 (5.4)	7.9 (5.4)
<5 years (n (%))	6089 (29.0)	21 003 (37.9)
5–10 years (n (%))	7296 (34.7)	19 414 (35.0)
>10 years (n (%))	7623 (36.3)	15 064 (27.2)
Number of concurrent diabetes medications	1.7 (0.8)	1.4 (0.7)
HbA1c (% (mmol/mol))	78.2 (16.7)	73.1 (16.6)
<8.0 (<64)(n (%))	4401 (20.9)	19 067 (34.4)
8.0–9.5 (64–80) (n (%))	8547 (40.7)	21 665 (39.0)
>9.5 (>80) (n (%))	8060 (38.4)	14 749 (26.6)
eGFR (mL/min)	87.9 (15.2)	82.7 (17.5)
45–<60 mL/min (n (%))	890 (4.2)	6997 (12.6)
60–90 (n (%))	9674 (46.0)	27 245 (49.1)
>90 (n (%))	10 444 (49.7)	21 239 (38.3)
BMI (kg/m^2^)	34.4 (6.7)	32.7 (6.6)
<25 kg/m^2^ (n (%))	946 (4.5)	5156 (9.3)
25–30 kg/m^2^ (n (%))	4747 (22.6)	16 040 (28.9)
>30 kg/m^2^ (n (%))	15 311 (72.9)	34 275 (61.8)
Previous genital mycosis (n (%))		
Never	16 877 (80.3)	46 663 (84.1)
<1 year	722 (3.4)	1753 (3.2)
1–5 years	1363 (6.5)	2943 (5.3)
>5 years	2046 (9.7)	4122 (7.4)
Corticosteroid use (n (%))	2317 (4.2)	718 (3.4)
Oestrogen use (n (%))	665 (1.2)	252 (1.2)
Immunomodulator use (n (%))	629 (1.1)	219 (1.0)

All values shown are mean (SD) unless otherwise stated.

BMI, body mass index; DPP4i, Dipeptidyl peptidase-4 inhibitor; eGFR, estimated glomerular filtration rate; SGTL2, sodium-glucose co-transporter-2.

### Women and those with prior infection have higher genital infection risk

**Figure 1:**
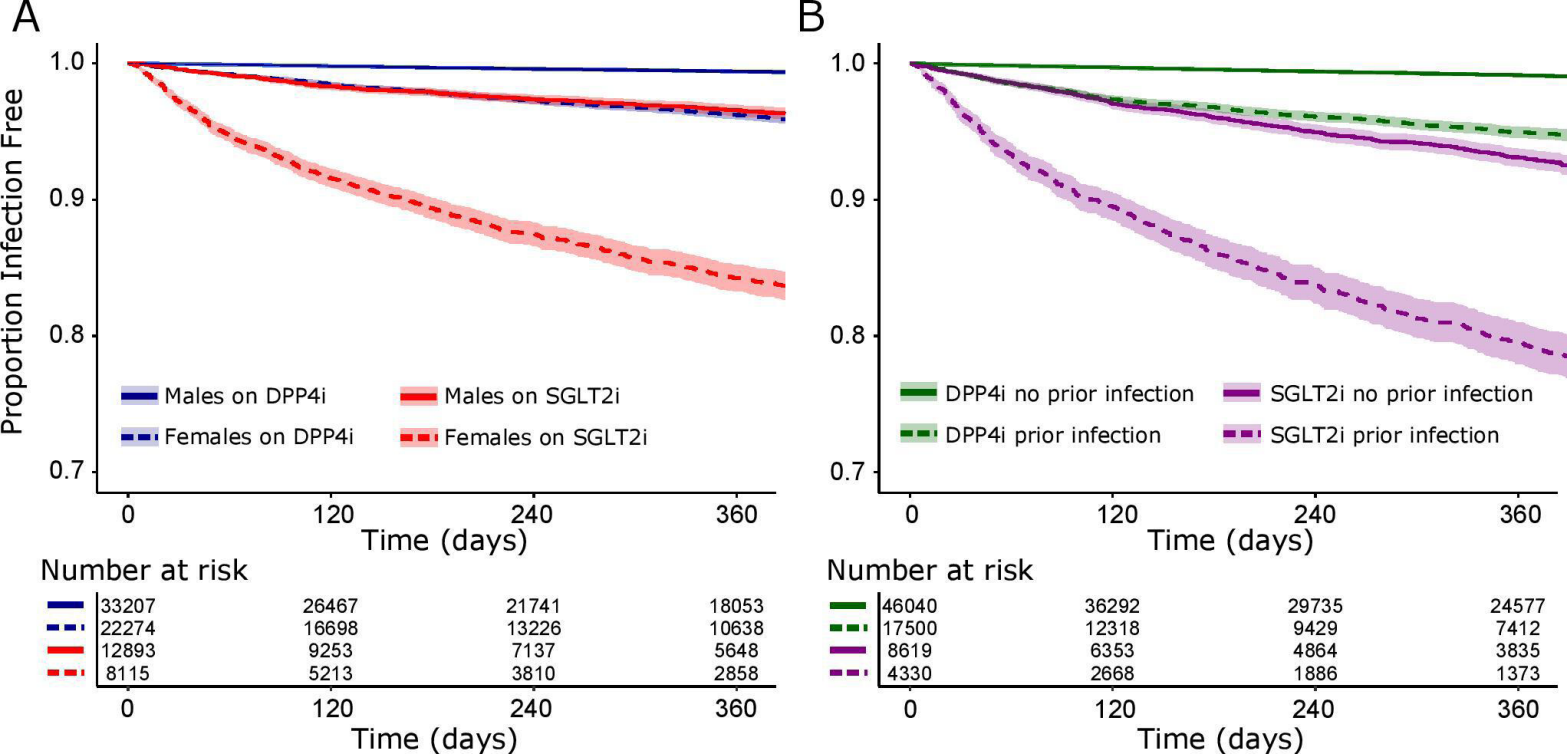
The proportion of people free from genital infection since drug initiation (A) by sex and medication class (SGLT2i and DPP4i) and (B) by history of prior genital infection and medication class. Number of events by group: (A) DPP4 inhibitor males n=228, DPP4 inhibitor females n=914, SGLT2i males n=371, SGLT2i females n=1092, (B) DPP4 inhibitor no prior infection n=484, DPP4 inhibitor and prior infection n=658, SGLT2i no prior infection n=729, SGLT2i and prior infection n=734. The shaded area represents the 95% CI. DPP4i, dipeptidyl peptidase-4 inhibitor; SGTL2i, sodium-glucoseco-transporter-2 inhibitor.

Genital infections were more common within the first year of treatment in those on SGLT2i (8.1%; 95% CI 7.6% to 8.5%) compared with DPP4i (1.8%; 95% CI 1.7% to 1.9%). Females were more likely to have a genital infection than males (figure 1A). Those with a history of previous genital infection were more likely to experience another infection after treatment initiation (figure 1B).

### High HbA_1c_ is not an additional risk factor for genital infection with SGLT2is

**Figure 2:**
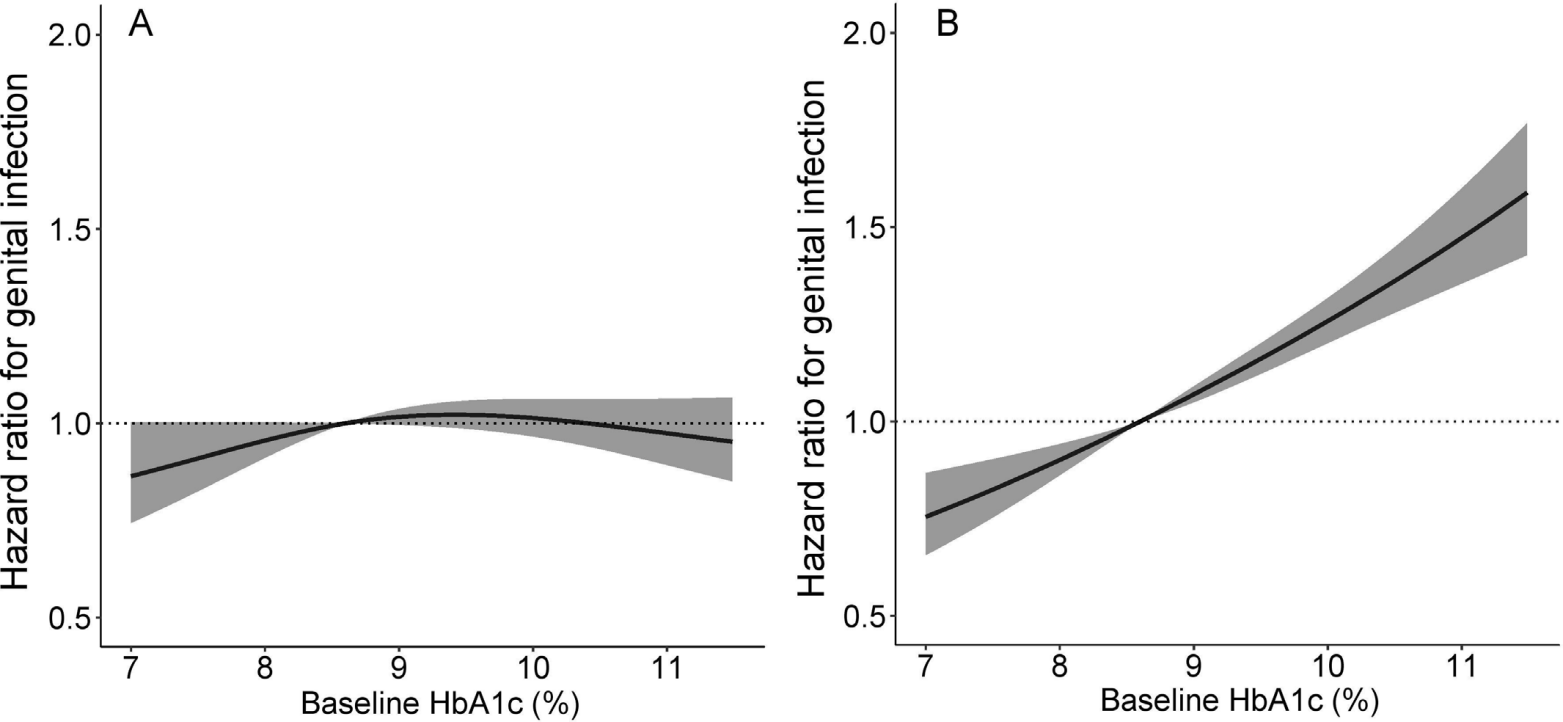
The association between HbA1c prior to medication initiation and genital infection after initiation of (A) an SGLT2 inhibitor and (B) a DPP4 inhibitor. Analyses are adjusted for age, gender, duration of diabetes, eGFR, BMI, and prior history of infection. The shaded area represents the 95% CI. A median HbA1c value of 8.6% (71mmol/mol) is used as the reference point. BMI, body mass index; DPP4i, dipeptidyl peptidase-4 inhibitor; eGFR, estimated glomerular filtration rate; SGTL2, sodium-glucoseco-transporter-2.

In multivariable models, female gender, higher BMI, and genital infection were independently associated with risk for future genital infection in people treated with both SGLT2is and DPP4is (table 2). The more recent the previous genital infection, the greater the subsequent infection risk. Higher HbA_1c_ was associated with greater infection risk in people using DPP4is but not SGLT2is (table 2 and figure 2). Sensitivity analysis using matched subgroups demonstrated the same pattern of association (online supplementary tables S2 and S3), as did analysis excluding those with non-specific diagnosis codes (online supplementary table S4).

**Table 2:** Associations between patient characteristics at medication initiation and subsequent genital infections with SGLT2 and DPP4 inhibitors

	SGLT2i (n=21 008) HR (95% CI) P value	DPP4i (n=55 481) HR (95% CI) P value
Age (years)	0.99 (0.99 to 1.00) p=0.069	0.98 (0.98 to 0.99) p<0.001
Female	3.66 (3.24 to 4.13) p<0.001	4.05 (3.51 to 4.66) p<0.001
Duration of diabetes (Reference group: <5 years)		
5–10 years	0.87 (0.77 to 0.98) p=0.024	1.01 (0.89 to 1.14) p=0.91
>10 years	0.93 (0.81 to 1.06) p=0.24	1.05 (0.91 to 1.22) p=0.49
HbA1c (Reference group:<8.0% (<64 mmol/mol))		
8.0%–9.5% (64–80 mmol/mol)	1.11 (0.97 to 1.27) p=0.13	1.12 (0.98 to 1.27) p=0.108
>9.5% (>80 mmol/mol)	0.95 (0.83 to 1.09) p=0.49	1.48 (1.29 to 1.71) p<0.001
eGFR (Reference group:>90 mL/min)		
60–90 mL/min	0.78 (0.56 to 1.08) p=0.13	0.76 (0.61 to 0.96) p=0.019
<60 mL/min	1.01 (0.90 to 1.13) p=0.87	0.96 (0.85 to 1.09) p=0.57
BMI (Reference group: 25–30 kg/m^2^)		
<25 kg/m^2^	0.78 (0.55 to 1.10) p=0.15	0.70 (0.53 to 0.93) p=0.016
>30 kg/m^2^	1.22 (1.07 to 1.39) p=0.005	1.29 (1.13 to 1.48) p<0.001
Previous genital mycosis (Reference group: no previous infection)		
<1 year	4.47 (3.81 to 5.24) p<0.001	8.90 (7.67 to 10.34) p<0.001
1–5 years	3.04 (2.64 to 3.51) p<0.001	4.13 (3.54 to 4.82) p<0.001
>5 years	1.77 (1.53 to 2.04) p<0.001	2.00 (1.68 to 2.38) p<0.001
C-statistic	0.76	0.82

BMI, body mass index; DDP4i, dipeptidyl peptidase-4 inhibitor; eGFR, estimated glomerular filtration rate; HR, hazard ratio; SGTL2, sodium-glucose co-transporter-2.

Including the use of oestrogens, corticosteroids, and other immune-modulating medications did not significantly modify the associations identified (online supplementary table S5). The use of these medications at treatment initiation was not associated with significantly increased risk for infection in the SGLT2i group but corticosteroid and oestrogen use was associated with increased risk in those initiating DPP4is.

### Future genital infection risk can be stratified using gender and infection history

**Figure 3:**
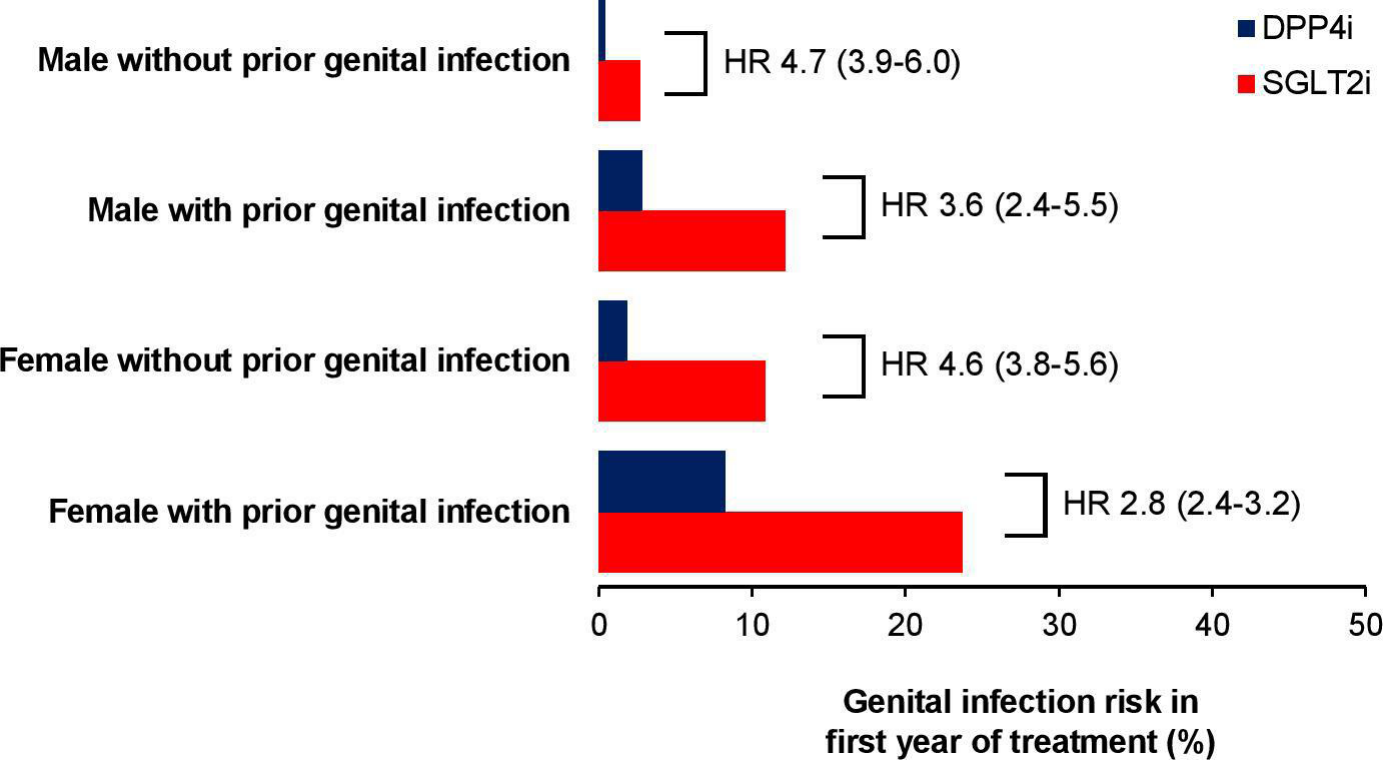
The absolute and relative risk of having a genital infection with an SGLT2i versus DPP4i by risk group. HRs are derived from propensity matched cohorts (online supplementary table S2) and are adjusted for age, gender, duration of diabetes, HbA_1c_, eGFR, BMI, and prior history of infection (online supplementary table S8). Numbers in brackets are 95% CIs. BMI, body mass index; DPP4i, dipeptidyl peptidase-4 inhibitor; eGFR, estimated glomerular filtration rate; HR, hazard ratio; SGTL2i, sodium-glucoseco-transporter-2 inhibitor.

Using the results of the regression models, we defined four key risk groups: males without a history of genital infection, males with a history of genital infection, females without a history of genital infection, females with a history of genital infection. These groups can be used to estimate the absolute risk for genital infection over the first year of treatment with an SGLT2i compared with a DPP4i (figure 3 and online supplementary table S6) and over longer durations (online supplementary table S7) and the HR for infection associated with using an SGLT2i compared with a DPP4i (figure 3 and online supplementary table S8).

### Genital infections are associated with a greater risk of discontinuation

Genital infections occurring within the first month of treatment were associated with a greater adjusted risk for subsequent discontinuation with both SGLT2i (19.4% without discontinued versus 31.8% with by 1 year; adjusted HR 1.48; 95% CI 1.21 to 1.81) and DPP4i (19.0% without versus 36.4% with by 1 year; adjusted HR 1.58; 95% CI 1.21 to 2.07).

## Conclusion

Our study shows sex and history of genital infection can be used to stratify the risk of genital infections over the first year of SGLT2i treatment. Absolute risk is markedly higher in those with prior infection (females 23.7%, males 12.1%), compared with those without (females 10.8%, males 2.7%). In contrast, we found no evidence of an association between high HbA_1c_ levels and increased risk of infection with SGLT2i, though we did for DPP4i. Early genital infection was associated with an increased discontinuation risk with SGLT2is (HR 1.48; 1.21 to 1.80); this effect was also observed with DPP4is (HR 1.58; 1.2 to 2.1).

### Comparison with the literature

Our findings suggest that the prevalence of genital infections in clinical practice in people using SGLT2is is slightly higher overall (8.1%; 95% CI 7.6% to 8.5%) that that reported in large clinical trials with SGLT2is (2.5% to 6.5%).^11–13^ Despite the lower incidence in trials, meta-analyses of trial data show a 4–6-fold increased risk of genital infection with SGLT2i compared with placebo, consistent with our findings.^10 26^ Prior history of genital infection was also associated with a greater risk of further infection on SGLT2i treatment in clinical trials.^16^ This finding fits clinical experience.

The associations we identified between sex and prior history of infection are supported in a much smaller national audit of SGLT2i use from secondary care;^27^ however, multivariable analysis was not performed in this study. A US claims-based study also found similar sex associations.^28^ Our data provide the first estimates of absolute and relative genital infection risk with SGLT2i across clinical groups defined using simple patient characteristics. To our knowledge, no previous study has explored the independent association between HbA1c and genital infection with SGLT2i, analyzed over a wide range of HbA_1c_ values and this is a critical and novel finding. We also provide the first simple clinical stratification tool to estimate genital infection risk across four key risk groups.

### Strengths and limitations

Important strengths of our analysis are the large cohort size, high levels of data completeness in the diabetes cohort from which our sample was derived and the real-world nature of the data. These factors mean our results have broad generalizability and can be applied to current clinical practice. There are some noteworthy limitations. Our analysis relies, in part, on correct clinical coding of genital infections and is therefore subject to inaccuracies resulting from non-coding or miscoding of genital infections. However, the rates of genital infections in our analysis are consistent with other smaller studies which used case note review to identify genital infections and therefore not subject to coding errors.^29 30^ Topical antifungal treatments are available to purchase ‘over-the-counter’ and therefore some people may have treated their genital infections without presenting to primary care; we therefore may have systematically under recorded infection rates. The severity of infections was not available; for example, paraphimosis and other complication data were not sufficiently well recorded to analyze these outcomes. An additional limitation is that data on the reasons for treatment discontinuation were not available for this study.

### Clinical implications

We provide the first simple clinical method for stratifying risk for genital infections with SGLT2i across four key risk groups; both absolute risks and HRs compared with DPP4i. This can be used in clinical practice to support patients to make more informed treatment selection decisions. If those at highest risk elect to start an SGLT2i, then practitioners should pay particular attention to counseling regarding genital hygiene and when to start antifungal treatments. While early infection was associated with a greater chance of discontinuation, this effect was also seen with DPP4i. Other recent work has demonstrated that simple clinical features can be used to stratify risk of side-effects with sulfonylurea and thiazolidinedione treatment.^31 32^ Along this, our findings provide a starting point for a more personalized approach to selecting type 2 diabetes treatment after metformin, with specific medication targeted according to individuals or subgroups based on their biological or risk characteristics.^33^

Interestingly, we found that people with high HbA_1c_ are not at additional genital infection risk with SGLT2i. This may be because glycosuria is a universal feature of SGLT2i treatment, occurring even in people with a normal HbA_1c_.^34^ It is therefore plausible that the association between glycosuria and genital infection reaches a saturation point above which increased glycosuria does not increase infection risk. This threshold may be exceeded with SGLTi use even in those with a relatively low HbA_1c_. By contrast, in those on DPP4i treatment, the amount of glycosuria will be low in those with the lowest serum glucose and will progressively increase with higher HbA1c as the serum glucose progresses past the renal threshold of maximum glucose resorption.^35^ It is similarly interesting that corticosteroids, immune-modulating medications, and oestrogen therapies were not associated with significant additional infection risk in those using SGLT2i. The reasons for this are unclear. While we are likely to be underpowered (particularly with immune-modulating therapies) to detect small increases in risk, our data reassuringly suggest no substantial increase in risk if these treatments are used concurrently with SGLT2i.

In conclusion, genital infections are common in people on SGLT2i in clinical practice. Major risk factors of infection are female sex and prior infection but not baseline HbA_1c_, meaning risk can be stratified by these two simple clinical features.
