# Supplementary material for: Risk factors for genital infections in people initiating SGLT2 inhibitors and their impact on discontinuation
Source: BMJ Open Diabetes Res Care. 2020 May 24;8(1):e001238. doi: 10.1136/bmjdrc-2020-001238 (PMC7252998; doi:10.1136/bmjdrc-2020-001238)
Supplement: Supplementary data [file bmjdrc-2020-001238supp001.pdf]

Risk factors for genital infections in people initiating SGLT2 inhibitors and their impact on discontinuation

Risk factors for genital infections in people initiating SGLT2 inhibitors and their impact on discontinuation: Supplementary appendix

Andrew P McGovern, Michael Hogg, Beverley M Shields, Naveed A Sattar, Rury R Holman, Ewan Pearson, Andrew Hattersley, Angus Jones, John Dennis, On behalf of the MASTERMIND Consortium

Additional collaborators in the MASTERMIND consortium:

William E Henley (PhD) – University of Exeter, UK

Mike Lonergan (PhD) – University of Dundee, UK

Lauren R Rodgers (PhD) – University of Exeter, UK

Willie T Hamilton (PhD) – University of Exeter, UK

Catherine Angwin (MSc) – University of Exeter, UK

Kennedy J Cruickshank (MD) – Kings College, London, UK

Andrew J Farmer (DM) – University of Oxford, UK

Stephen C L Gough (MD) – University of Oxford, UK

Alastair M Gray (PhD) – University of Oxford, UK

Christopher Hyde (MD) – University of Exeter, UK

Christopher Jennison (PhD) – University of Bath, UK

Mark Walker (MD) – University of Newcastle, UK

Risk factors for genital infections in people initiating SGLT2 inhibitors and their impact on discontinuation

Supplementary tables

DPP4 inhibitors (n=55,481)	n (%)
**Alogliptin**	3,662 (6.6)
**Linagliptin**	6,636 (12.0)
**Saxagliptin**	6,043 (10.9)
**Sitagliptin**	37,173 (67.0)
**Vildagliptin**	1,967 (3.5)
**SGLT2 inhibitors (n=21,008)**	**n (%)**
**Canagliflozin**	3,292 (15.7)
**Dapagliflozin**	11,731 (55.8)
**Empagliflozin**	5,985 (28.5)

Table S1. Medications initiated within each medication class.

Risk factors for genital infections in people initiating SGLT2 inhibitors and their impact on discontinuation

	SGLT2 inhibitor (n=19,359)	DPP4 inhibitor (n=19,359)	p
**Age (years)**	60.5 (10.2)	60.8 (9.3)	0.013
**Female [n (%)]**	7,457 (38.5)	7,512 (38.8)	0.573
**Duration of diabetes (years)**	8.8 (5.6)	8.7 (5.2)	0.079
**<5 years [n (%)]**	6,144 (31.7)	5,993 (31.0)	0.224
**5-10 years [n (%)]**	6,841 (35.3)	6,878 (35.5)	
**>10 years [n (%)]**	6,374 (32.9)	6,488 (33.5)	
**Number of concurrent diabetes medications**	1.6 (0.6)	1.6 (0.8)	0.003
**HbA1c [% (mmol/mol)]**	76.8 (16.8)	77.5 (16.6)	<0.001
**<8.0 (<64) [n (%)]**	4,483 (23.2)	4,344 (22.4)	0.234
**8.0-9.5 (64-80) [n (%)]**	7,982 (41.2)	8,034 (41.5)	
**>9.5 (>80) [n (%)]**	6,894 (35.6)	6,981 (36.1)	
**eGFR (ml/min)**	87.3 (16.1)	87.5 (15.2)	0.281
**45 - <60 ml/min [n (%)]**	939 (4.9)	890 (4.6)	0.431
**60 - 90 [n (%)]**	9,009 (46.5)	9,086 (46.9)	
**>90 [n (%)]**	9,411 (48.6)	9,383 (48.5)	
**BMI (kg/m^2)**	33.8 (6.5)	34.2 (6.7)	<0.001
**<25 kg/m^2 [n (%)]**	941 (4.9)	945 (4.9)	0.066
**25-30 kg/m^2 [n (%)]**	4,469 (23.1)	4,661 (24.1)	
**>30 kg/m^2 [n (%)]**	13,949 (72.1)	13,753 (71.0)	
**Previous genital mycosis [n (%)]**			
**Never**	15,699 (81.1)	15,851 (81.9)	0.161
**<1 year**	675 (3.5)	677 (3.5)	
**1-5 years**	1,188 (6.1)	1,148 (5.9)	
**>5 years**	1,797 (9.3)	1,683 (8.7)	

Table S2. The baseline characteristics of propensity matched groups of people initiated on SGLT2 inhibitors and DPP4 inhibitors. All values shown are mean (SD) unless otherwise stated. eGFR = estimated glomerular filtration rate, BMI = body mass index.

Risk factors for genital infections in people initiating SGLT2 inhibitors and their impact on discontinuation

	SGLT2 inhibitor (n= 19,359) HR (95% CI) p value	DPP4 inhibitor (n= 19,359) HR (95% CI) p value
**Age (years)**	0.992 (0.985, 0.999) p = 0.023	0.984 (0.973, 0.995) p = 0.005
**Female**	3.70 (3.27, 4.20) p < 0.001	4.12 (3.29, 5.15) p < 0.001
**Duration of diabetes [Reference group: < 5 years]**		
**5 to 10 years**	0.89 (0.79, 1.01) p = 0.077	1.01 (0.83, 1.24) p = 0.903
**>10 years**	1.01 (0.87, 1.16) p = 0.94	0.98 (0.77, 1.24) p = 0.86
**HbA1c [Reference group: <8.0% (<64 mmol/mol)]**		
**8.0-9.5% (64-80 mmol/mol)**	1.11 (0.97, 1.27) p = 0.14	1.16 (0.91, 1.47) p = 0.25
**>9.5% (>80 mmol/mol)**	0.98 (0.85, 1.13) p = 0.75	1.46 (1.15, 1.86) p = 0.003
**eGFR [Reference group: >90 ml/min]**		
**60-90 ml/min**	0.76 (0.55, 1.05) p = 0.092	0.63 (0.35, 1.15) p = 0.14
**<60 ml/min**	1.00 (0.89, 1.13) p = 0.97	1.04 (0.85, 1.27) p = 0.71
**BMI [Reference group: 25-30 kg/m^2]**		
**< 25 kg/m^2**	0.78 (0.55, 1.10) p = 0.15	0.63 (0.34, 1.18) p = 0.15
**> 30 kg/m^2**	1.26 (1.10, 1.44) p = 0.001	1.15 (0.91, 1.45) p = 0.24
**Previous genital mycosis [Reference group: no previous infection]**		
**<1 year**	4.32 (3.67, 5.10) p < 0.001	9.85 (7.79, 12.45) p < 0.001
**1-5 years**	3.03 (2.61, 3.52) p < 0.001	4.20 (3.30, 5.35) p < 0.001
**>5 years**	1.76 (1.51, 2.06) p < 0.001	1.95 (1.49, 2.56) p < 0.001
**C-statistic**	0.75	0.81

Table S3. Associations between patient characteristics at medication initiation and subsequent development of genital infections during medication use with SGLT2 and DPP4 inhibitors in propensity matched cohorts.

Risk factors for genital infections in people initiating SGLT2 inhibitors and their impact on discontinuation

	SGLT2 inhibitor (n= 8,115) HR (95% CI) p value	DPP4 inhibitor (n= 22,271) HR (95% CI) p value
**Age (years)**	0.992 (0.985, 0.999) p = 0.023	0.984 (0.973, 0.995) p = 0.005
**Female**	3.70 (3.27, 4.20) p < 0.001	4.12 (3.29, 5.15) p < 0.001
**Duration of diabetes [Reference group: < 5 years]**		
**5 to 10 years**	0.89 (0.79, 1.01) p = 0.077	1.01 (0.83, 1.24) p = 0.903
**>10 years**	1.01 (0.87, 1.16) p = 0.940	0.98 (0.77, 1.24) p = 0.860
**HbA1c [Reference group: <8.0% (<64 mmol/mol)]**		
**8.0-9.5% (64-80 mmol/mol)**	1.11 (0.97, 1.27) p = 0.14	1.16 (0.91, 1.47) p = 0.25
**>9.5% (>80 mmol/mol)**	0.98 (0.85, 1.13) p = 0.75	1.46 (1.15, 1.86) p = 0.003
**eGFR [Reference group: >90 ml/min]**		
**60-90 ml/min**	0.76 (0.55, 1.05) p = 0.092	0.63 (0.35, 1.15) p = 0.14
**<60 ml/min**	1.00 (0.89, 1.13) p = 0.97	1.04 (0.85, 1.27) p = 0.71
**BMI [Reference group: 25-30 kg/m^2]**		
**< 25 kg/m^2**	0.78 (0.55, 1.10) p = 0.15	0.63 (0.34, 1.18) p = 0.15
**> 30 kg/m^2**	1.26 (1.10, 1.44) p = 0.001	1.15 (0.91, 1.45) p = 0.24
**Previous genital mycosis [Reference group: no previous infection]**		
**<1 year**	4.32 (3.67, 5.10) p < 0.001	9.85 (7.80, 12.45) p < 0.001
**1-5 years**	3.03 (2.61, 3.52) p < 0.001	4.20 (3.30, 5.35) p < 0.001
**>5 years**	1.76 (1.51, 2.06) p < 0.001	1.95 (1.49, 2.56) p < 0.001
**C-statistic**	0.67	0.79

Table S4. A sensitivity analysis of the associations between patient characteristics at medication initiation and subsequent development of genital infections* during medication use with SGLT2 and DPP4 inhibitors. *for this analysis genital infections are defined as either genital infection specific treatment (e.g. antifungal vaginal pessary) or genital specific codes (e.g. vaginal candidiasis); cases identified by the combination of a non-specific diagnosis (i.e. "thrush") and a concurrent prescription for a topical steroid are not included. Only women are included in this analysis as there are no genital specific treatments for men and few genital specific diagnosis codes available.

Risk factors for genital infections in people initiating SGLT2 inhibitors and their impact on discontinuation

	SGLT2 inhibitor (n=21,008) HR (95% CI) p value	DPP4 inhibitor (n=55,481) HR (95% CI) p value
**Age (years)**	0.993 (0.986, 0.999) p = 0.031	0.983 (0.977, 0.990) p < 0.001
**Female**	3.71 (3.29, 4.19) p < 0.001	3.97 (3.44, 4.57) p < 0.001
**Duration of diabetes [Reference group: < 5 years]**		
**5 to 10 years**	0.88 (0.77, 0.99) p = 0.034	1.01 (0.90, 1.15) p = 0.832
**>10 years**	0.94 (0.83, 1.08) p = 0.379	1.08 (0.93, 1.25) p = 0.344
**HbA1c [Reference group: <8.0% (<64 mmol/mol)]**		
**8.0-9.5% (64-80 mmol/mol)**	1.10 (0.96, 1.26) p = 0.161	1.12 (0.98, 1.27) p = 0.109
**>9.5% (>80 mmol/mol)**	0.94 (0.82, 1.08) p = 0.398	1.49 (1.30, 1.72) p < 0.001
**eGFR [Reference group: >90 ml/min]**		
**60-90 ml/min**	0.77 (0.56, 1.06) p = 0.107	0.75 (0.60, 0.94) p = 0.012
**<60 ml/min**	1.00 (0.90, 1.12) p = 0.962	0.96 (0.84, 1.09) p = 0.504
**BMI [Reference group: 25-30 kg/m^2]**		
**< 25 kg/m^2**	0.79 (0.56, 1.11) p = 0.179	0.71 (0.53, 0.95) p = 0.021
**> 30 kg/m^2**	1.18 (1.03, 1.35) p = 0.016	1.28 (1.12, 1.46) p < 0.001
**Previous genital mycosis [Reference group: no previous infection]**		
**<1 year**	4.42 (3.76, 5.20) p < 0.001	8.59 (7.39, 9.99) p < 0.001
**1-5 years**	3.03 (2.63, 3.49) p < 0.001	4.04 (3.46, 4.71) p < 0.001
**>5 years**	1.76 (1.52, 2.04) p < 0.001	1.99 (1.67, 2.37) p < 0.001
**Corticosteroid use**	1.14 (0.91, 1.43) p = 0.256	1.33 (1.07, 1.65) p = 0.011
**Oestrogen use**	0.91 (0.66, 1.25) p = 0.567	1.80 (1.40, 2.31) p < 0.001
**Immunomodulator use**	0.82 (0.51, 1.30) p = 0.398	1.20 (0.80, 1.80) p = 0.380
**C-statistic**	0.76	0.82

Table S5. Associations between patient characteristics at medication initiation and subsequent development of genital infections during medication use with SGLT2 and DPP4 inhibitors with the inclusion of baseline use of corticosteroids, oestrogen containing therapies, and immune-modulating medications.

Risk factors for genital infections in people initiating SGLT2 inhibitors and their impact on discontinuation

Risk Group	SGLT2 inhibitor (n=21,008)			DPP4 inhibitor (n=55,481)		
	n	Events	Absolute risk (%) (95% CI)	n	Events	Absolute risk (%) (95% CI)
Males without a history of genital infection	11,845	275	2.7 (2.3, 3.1)	31,296	165	0.4 (0.4, 0.5)
Males with a history of genital infection	1,048	96	12.1 (9.6, 14.6)	1,911	63	2.8 (1.9, 3.7)
Females without a history of genital infection	5,032	454	10.8 (9.8, 11.8)	15,367	319	1.8 (1.6, 2.1)
Females with a history of genital infection	3,083	638	23.7 (21.8, 25.5)	6,907	595	8.2 (7.4, 9.0)

Table S6. The risk of developing a genital infection within one year after initiating an SGLT2 or DPP4 inhibitor across the four selected key clinical risk groups.

Risk factors for genital infections in people initiating SGLT2 inhibitors and their impact on discontinuation

Risk Group	SGLT2 inhibitor Absolute risk (%) (95% CI)			DPP4 inhibitor Absolute risk (%) (95% CI)		
	2 years	3 years	4 years	2 years	3 years	4 years
Males without a history of genital infection	3.8 (3.4, 4.3)	5.4 (4.6, 6.1)	5.7 (4.9, 6.5)	0.8 (0.7, 0.9)	1.2 (1.0, 1.4)	1.4 (1.2, 1.7)
Males with a history of genital infection	16.8 (15.2, 18.3)	20.7 (18.7, 22.8)	23.7 (20.8, 26.6)	3.4 (3.0, 3.8)	4.7 (4.1, 5.2)	5.8 (5.1, 6.5)
Females without a history of genital infection	17.2 (13.8, 20.5)	18.7 (14.7, 22.5)	18.7 (14.7, 22.5)	5.5 (4.1, 7.0)	7.6 (5.6, 9.6)	9.1 (6.6, 11.5)
Females with a history of genital infection	34.2 (31.5, 36.7)	41.6 (38.1, 44.9)	46.4 (41.8, 50.7)	13.8 (12.5,15.0)	17.8 (16.2, 19.3)	21.3 (19.3, 23.2)

Table S7. The cumulative risk of developing a genital infection within at two, three and four years after initiating an SGLT2 or DPP4 inhibitor across the four selected key clinical risk groups

Risk factors for genital infections in people initiating SGLT2 inhibitors and their impact on discontinuation

	Males, no prior infection (n=22,044) HR (95% CI) p value	Males, prior infection (n=1,704) HR (95% CI) p value	Females, no prior infection (n=9,506) HR (95% CI) p value	Females, prior infection (n=5,464) HR (95% CI) p value
**Age (years)**	0.98 (0.97, 1.00) p = 0.007	0.99 (0.97, 1.01) p = 0.591	0.98 (0.97, 0.99) p = 0.002	1.00 (0.99, 1.01) p = 0.495
**Duration of diabetes [Reference group: < 5 years]**				
**5 to 10 years**	0.77 (0.60, 0.98) p = 0.038	1.56 (0.98, 2.48) p = 0.064	0.88 (0.73, 1.06) p = 0.181	0.95 (0.81, 1.12) p = 0.559
**>10 years**	0.86 (0.66, 1.14) p = 0.296	1.16 (0.67, 2.03) p = 0.595	0.98 (0.79, 1.21) p = 0.854	1.06 (0.89, 1.28) p = 0.512
**HbA1c [Reference group: <8.0% (<64 mmol/mol)]**				
**8.0-9.5% (64-80 mmol/mol)**	0.93 (0.73, 1.29) p = 0.580	1.36 (0.78, 2.39) p = 0.279	1.17 (0.95, 1.43) p = 0.137	1.16 (0.95, 1.40) p = 0.139
**>9.5% (>80 mmol/mol)**	0.71 (0.54, 0.94) p = 0.017	0.79 (0.44, 1.41) p = 0.424	1.32 (1.07, 1.63) p = 0.011	1.13 (0.94, 1.37) p = 0.201
**eGFR [Reference group: >90 ml/min]**				
**60-90 ml/min**	0.65 (0.32, 1.35) p = 0.253	1.01 (0.35, 2.93) p = 0.980	0.75 (0.47, 1.21) p = 0.240	0.70 (0.45, 1.10) p = 0.123
**<60 ml/min**	0.93 (0.74, 1.18) p = 0.565	0.95 (0.62, 1.46) p = 0.818	1.04 (0.87, 1.24) p = 0.674	1.06 (0.91, 1.23) p = 0.490
**BMI [Reference group: 25-30 kg/m^2]**				
**< 25 kg/m^2**	0.37 (0.15, 0.91) p = 0.030	0.89 (0.27, 2.93) p = 0.844	0.93 (0.58, 1.49) p = 0.758	0.77 (0.48, 1.25) p = 0.294
**> 30 kg/m^2**	1.21 (0.94, 1.54) p = 0.136	1.15 (0.74, 1.79) p = 0.527	1.32 (1.06, 1.63) p = 0.013	1.21 (1.00, 1.45) p = 0.046
**Previous genital mycosis [Reference group: >5 years]**				
**<1 year**	-	3.24 (1.92, 5.48) p < 0.001	-	3.28 (2.76, 3.91) p < 0.001
**1-5 years**	-	2.49 (1.59, 3.90) p < 0.001	-	1.83 (1.54, 2.16) p < 0.001
**SGLT2 inhibitor [Reference group: DPP4 inhibitor]**	4.67 (3.65, 5.98) p < 0.001	3.61 (2.39, 5.45) p < 0.001	4.58 (3.78, 5.55) p < 0.001	2.79 (2.42, 3.22) p < 0.001
**C-statistic**	0.72	0.72	0.69	0.71

Table S8. Associations between the selection of SGLT2 or DPP4 inhibitors initiation and subsequent development of genital infections during medication use with adjustment for patient characteristics at baseline in the four key risk groups.
